# Early Prophylactic Hypothermia for Patients With Severe Traumatic Injury: Premature to Close the Case

**DOI:** 10.3389/fneur.2019.00344

**Published:** 2019-04-09

**Authors:** Shan Min Chin, Didier Wion

**Affiliations:** INSERM UMR1205, Faculté Médecine Pharmacie, Université Grenoble Alpes, La Tronche, France

**Keywords:** therapeutic hypothermia, traumatic brain injury, focal hypothermia, pro-resolving therapies, specialized proresolving mediators

## Background

In a recent well-designed and executed clinical trial, Cooper and collaborators found no evidence to support the use of early prophylactic hypothermia in patients with severe traumatic brain injury (TBI) (1). This study demonstrates that there is no role for the initiation of hypothermia during the acute phase of TBI (1, 2). However, it would be damaging to abandon the concept prematurely.

## Inflammation Also Paves the Way to Tissue Repair

As soon as trauma occurs, the inflammatory cascade begins to take place. The deleterious role of inflammation in the secondary injury response is well-documented, hence the rationale to attempt early prophylactic hypothermia in TBI. However, inflammation also initiates tissue repair and regeneration (3–6). We now know that the secondary injury response accompanies the regenerating and healing response. Rather than considering the injury cascades and resolving phases as two sequential antagonistic processes, we now understand them as two overlapping aspects of the same dynamic process dedicated to regeneration and healing (3–5). The resolving phase is an active process intrinsic to the progression of the inflammatory response. It is triggered by several pro-inflammatory mediators such as IL-1β and TNF-α, that recruit, among others, IL-10, TGFβ, and the members of a novel superfamily of lipid mediators called specialized pro-resolving mediators (SPMs) (3–6). The members of the SPMs family that include lipoxins, resolvins (Rvs), maresins, and protectins are central to this process (3, 7). They actively terminate inflammation and promote tissue regeneration (3, 7, 8). Indeed, the temporal switch from the generation of proinflammatory mediators to the synthesis of SPMs is essential to the resolution of inflammation and healing (3, 7, 8). Several SMPs have already been detected in human brain (9). As regards TBI, at least two members of the SPM family, namely Resolvin D1 and Protectin D1are identified in mouse brain tissue following stroke-injury (10). Recent experiments performed on rat also show that Resolvin D1 decreases remote neuro-inflammation and improves functional recovery after focal brain damage (11). Resolvin D2 also protects against cerebral ischemia/reperfusion injury (12).

Because the synthesis of SPMs is intrinsic to the inflammatory response, any uncontrolled braking of the inflammatory process can impair their synthesis, that would in turn affect the resolving phase and brain healing (3, 5–7). This could explain the paradoxical “resolution-toxic” side effects that some anti-inflammatory drugs or therapies may have (3, 5, 13). In this emerging framework, where the inflammatory response is both a target and a tool for therapy (3, 5, 7, 14), many outstanding questions remain. For examples, how does prophylactic hypothermia affect the complex network of proinflammatory and proresolving mediators and thus the resolving phase of inflammation? More generally, to what extent does hypothermia delay the synthesis of SPMs and of other resolving factors or neuroprotective cytokines and impairs their function? Does the process of cooling and rewarming hinder the prophylactic potential of hypothermia by desynchronizing the resolving phase? What happens to the clearance of apoptotic cells and cell debris during and after hypothermia? Currently, we are still missing the answers to those questions. It would be rather surprising if hypothermia did not impair or delay some processes involved in tissue repair and regeneration. Combining a pro-resolving therapeutic strategy may be the necessary step to reveal the prophylactic potential of hypothermia in TBI.

## Focal Hypothermia: the Alternative to Whole-Body Cooling Protocols

Another concern is the whole-body cooling protocol used in clinical trials. The potential complications of systemic hypothermia are well-known. They mainly include infection like pneumonia, and hypotension. Moreover, as regards the use of therapeutic hypothermia in TBI, a single optimal hypothermic temperature for all regions of the brain subjected to trauma probably does not exist. The effective prophylactic temperature is unlikely to be the same for the lesion core, the penumbra, and the remainder of the body. Importantly, these different optimal temperatures, once defined, will need to be finely tuned over time. We must handle hypothermia as a dynamic and local parameter. The alternative to whole-body cooling is focal hypothermia. It would allow deliverance of specific temperature gradients to local space-time frameworks without the side effects observed with whole-body hypothermia. The concept of focal brain hypothermia is not new. As early as 1940, T Fay tried to treat glioblastoma patients by implanting in the resection cavity metal capsules connected to refrigerated liquids (15). Nowadays, implantable focal cooling devices are in development to prevent epileptic seizures (16, 17) and glioma recurrence (18). In a model of experimental cerebral ischemia, neuroprotective effects are obtained in free-moving rats with an implantable focal brain cooling device (19). In non-human primate an epidural cooling to 15°C with a cooling pad achieves a brain temperature to 34–35°C at 15 mm deep and 28–32°C at 10 mm deep (20). The same brain surface temperature of about 15°C was also recently achieved in non-human primates with a chronically implanted titanium cooling plate (21). These preclinical studies point to an important limitation of our current focal surface devices. The diffusion of hypothermia in the brain parenchyma is limited to around 15 mm due to thermal buffering by blood circulation (22), and to the low thermal conductivity of the brain (23). Hence, epidural focal cooling should benefit only patients with superficial neocortical lesions. For deeper focal traumatic lesions, and as long as we will not have any method to increase the brain cooling diffusion or mini invasive implantable intracerebral cooling devices (17), selective endovascular intra-carotid infusion of cold saline remains the alternative to whole body cooling (24).

Clearly, there are still many research and technical challenges ahead (25). It is in response to these challenges that we need to develop implantable microdevices capable to continuously and locally adjusting the optimal gradients of hypothermia in conjunction with pro-resolving therapies.

## Concluding Remarks

The results of the trial conducted by Cooper et al showed that early hypothermia did not improve long-term favorable functional outcomes in patients with severe TBI. No effect of hypothermia on intracranial pressure is also found (1). However, it is premature to close the case of therapeutic hypothermia in TBI. Understanding the discrepancy between these results and the huge amount of convincing experimental evidence is now the clinical challenge we must overcome. Experimental evidence demonstrates that hypothermia can protect brain function by moderating immune inflammatory responses, reducing brain metabolism and edema. TBI is a highly heterogeneous condition, and not all the type of brain lesions would respond similarly to a standardized protocol of whole body cooling. In a certain way, we could say that the concept of therapeutic hypothermia is valid but that we do not know yet how to make it work in large randomized clinical trials. Patient stratification and the combination of therapeutic hypothermia with minimally invasive surgery and adjuvant therapies is probably necessary to reveal the therapeutic effects of hypothermia in RCTs. As regards the self-reinforcing cycle between inflammation and edema/intracranial pressure (26), we know that the anti-inflammatory effects of mild hypothermia combined with minimally invasive surgery can reduce brain edema (27, 28). Posttraumatic hypothermia also reduces the levels of MMP9 that is a major contributor to blood brain barrier disruption and edema (29, 30). Hence, therapeutic hypothermia remains a reasonable option in a multimodal approach as a rescue therapy for patients with poorly controlled intracranial pressure and/or cerebral edema. Another point to consider is the possibility to use therapeutic hypothermia to extend the therapeutic time window of some drugs (31).

There is a future for prophylactic hypothermia in TBI. It resides in patients stratification, and in the development of synergies between focal intelligent cooling devices, pro-resolving therapies (32, 33), and novel neuroprotective drugs (34).

## Author Contributions

All authors listed have made a substantial, direct and intellectual contribution to the work, and approved it for publication.

### Conflict of Interest Statement

The authors declare that the research was conducted in the absence of any commercial or financial relationships that could be construed as a potential conflict of interest.
